# Morocco's National Response to the COVID-19 Pandemic: Public Health Challenges and Lessons Learned

**DOI:** 10.2196/31930

**Published:** 2021-09-17

**Authors:** Abdelaziz Barkia, Hammou Laamrani, Abdelmounaim Belalia, Abderrahman Benmamoun, Yousef Khader

**Affiliations:** 1 Epidemic Diseases Service Ministry of Health Rabat Morocco; 2 Water, Energy, Food Security and Climate Change Nexus Economic Sector League of Arab States Cairo Egypt; 3 Ministry of Health Rabat Morocco; 4 Ministry of Health Mohammadia Morocco; 5 Department of Public Health Jordan University of Science and technology Irbid Jordan

**Keywords:** COVID-19, public health, challenges, prevention, control, infectious disease

## Abstract

This report aimed to provide an overview of the epidemiological situation of COVID-19 in Morocco and to review the actions carried out as part of the national response to this pandemic. The methodology adopted was based on literature review, interviews with officials and actors in the field, and remote discussion workshops with a multidisciplinary and multisectoral working group. Morocco took advantage of the capacities already strengthened within the framework of the application of the provisions of the International Health Regulations (IHR) of 2005. A SWOT analysis made it possible to note that an unprecedented political commitment enabled all the necessary means to face the pandemic and carry out all the response activities, including a campaign of relentless communication. Nevertheless, and despite the efforts made, the shortage of human resources, especially those qualified in intensive care and resuscitation, has been the main drawback to be addressed. The main lesson learned is a need to further strengthen national capacities to prepare for and respond to possible public health emergencies and to embark on a process overhaul of the health system, including research into innovative tools to ensure the continuity of the various disease prevention and control activities. In addition, response to a health crisis is not only the responsibility of the health sector but also intersectoral collaboration is needed to guarantee an optimal coordinated fight. Community-oriented approaches in public health have to be strengthened through more participation and involvement of nongovernmental organizations (NGOs) and civil society in operational and strategic planning.

## Introduction

### Background

Morocco, like all countries of the world, is facing an unprecedented situation of a global pandemic due to COVID-19 [1]. Since the announcement of the first alerts by the World Health Organization (WHO) relating to the emergence and spread of this new disease [2], the Moroccan government deployed a national monitoring and response plan adopting a spirit of solidarity and involving the public authorities and the whole of society.

A few days after the declaration of this first case on March 2, 2020 in Morocco and the notification of other cases, the “State of health emergency” was declared and a series of measures including containment was implemented to contain the spread of the virus [3]. Morocco has a population of around 36 million and is considered a middle-income country with limited health care capacity compared to many other countries in the region. However, the country has accumulated several experiences in the field of public health emergency management and has prepared relatively well to deal with the emergence of sanitary risks related to the new virus, especially through training programs and strengthening organizational and managerial capacities.

Although the crisis continues to be a challenge to society as a whole, much can be learned from the actions already undertaken so far. Therefore, evaluation and review of the implementation of the various health interventions must be considered as a continuous process [4]. This would make it possible to assess the effectiveness of the actions implemented as well as their coherence, consistency, and alignment with the International Health Regulations (IHR) 2005 and guidelines [5,6].

### Objectives

The main objective of this paper was to assess the actions undertaken in Morocco during the response to COVID-19 in order to draw lessons and identify good practices to capitalize on for better management of a potential new wave or future epidemics. Moreover, this study aimed to review and discuss the different interventions implemented as part of the national response plan against COVID-19; conduct an analysis of the strengths, weaknesses, opportunities, and threats (SWOT) of national preparedness and response to the COVID-19 pandemic; and discuss the main lessons learned from the national preparation and response to the pandemic.

## Methodological Approach

The present work was based on 3 research processes, namely a review of key documents published; interviews with managers, actors, and resource persons; and remote discussions with a multidisciplinary and multisectoral working group set up for this purpose. Raw data were collected by analyzing memos and epidemiological bulletins and by regularly consulting the website of the Ministry of Health. A daily follow-up of press articles and statements from various officials of the Ministry of Health and members of the government was further carried out. The research process begun with the announcement of the COVID-19 pandemic in late December 2019 and ended by October 2020.

The discussion group was made up of 12 participants including 4 former officials at the level of the Ministry of Health, 4 former managers and health professionals including 2 Field Epidemiology Training Program (FETP) graduates, 2 medical journalists, and 2 biomedical research professors. This discussion group focused on the SWOT analysis through 3 workshops organized remotely to collect opinions regarding the operational implementation of the actions planned on the ground within the framework of the national COVID-19 monitoring and response plan. The principal investigator facilitated the workshops.

For each theme, a direct question was asked about strengths and weaknesses; then, participants were asked to suggest the opportunities to strengthen the response to the pandemic and also the threats that may hamper its control.

## The Epidemiological Situation of COVID-19 in Morocco

The first case of COVID-19 in Morocco was detected on March 2, 2020. The first case was a 39-year-old man, originally from and living in Casablanca, who traveled to a European country and returned to Morocco on February 27, 2020. The first COVID-19–associated death was announced on March 12, 2020, and the first case of local transmission was recorded on March 13, 2020 [7,8].

**Figure 1:**
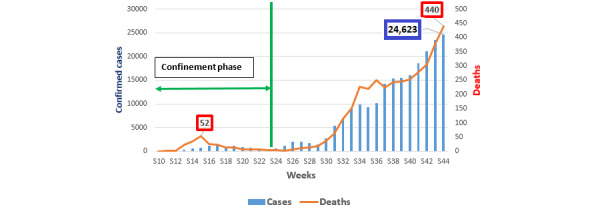
Weekly evolution of the number of COVID-19 cases and deaths in Morocco from March 2, 2020 to October 31, 2020.

Between March 2, 2020 and October 31, 2020, a cumulative 219,084 confirmed cases was recorded (ie, an average of 898 cases per day). The total number of deaths was 3695 with an average of 8 deaths per day. The case fatality rate at the end of October was 1.7%. The weekly evolution of cases and deaths (Figure 1) shows a gradual increase and then an exacerbation in the number of confirmed cases and deaths. The epidemiological situation of the disease evolved in 3 stages of development of the epidemic. The first phase was marked by control of the situation with few cases and deaths (phase corresponding to the lockdown period). The second phase, corresponding with the first gradual lifting of confinement, was marked by a significant, steady increase in the number of cases. The third phase, corresponding with a relatively generalized lifting of lockdown, was marked by an exacerbation in the number of new cases and deaths.

Thus, the evolution of the number of cases followed a geometric progression from the 3rd phase of the epidemic. Just after the feast of the sacrifice (Eid El Adha), a new situation was marked by an increase in the number of deaths and patients in intensive care and resuscitation unit with very strong pressure on the health care system. The highest number of cases and deaths was recorded in the last week of October 2020 (week 44 of the year) with 24,623 confirmed cases and 440 deaths.

Regarding the spatial distribution of cases, all 12 regions of Morocco were affected, with variable attack rates ranging from 5 per 100,000 inhabitants in the Fes-Meknes Region to 6 per 100,000 inhabitants in the region of Dakhla-Oued Eddahab located in the extreme south of Morocco. The cumulative incidence in the Casablanca-Settat region, which recorded the highest number of cases, was 3 per 100,000 inhabitants, while the national average was 4 per 100,000 inhabitants.

According to data made public by the Ministry of Health, among the cases detected from March 2, 2020 to September 21, 2020, asymptomatic cases on admission represented 74.9%, mild cases represented 14.1%, moderate cases represented 9.6%, severe cases represented 1%, and critical cases represented 0.4% [9].

## National Preparation and Response

### Preparedness and Coordination of the National Response

With few exceptions, the same structures responsible for coordinating the response against the influenza A (H1N1) 2009 pandemic have been reactivated to lead the response operations against COVID-19. A national plan for monitoring and responding to infection was officially launched on January 27, 2020. A high-level commission chaired by the head of government has been set up to take political, diplomatic, regulatory, cooperation, and response orientation decisions. The Ministry of Health has set up a steering committee for the health component of the response. The tasks of epidemiological monitoring and coordination have been entrusted to the National Public Health Emergency Operations Center as part of the operationalization of the actions included in the National Health Security Plan 2018-2022 [10].

A special fund for the management of the coronavirus pandemic “The COVID-19 Fund” has been created. This fund is earmarked to upgrade the medical services in terms of infrastructure and resources in an emergency, and it also aims to support the national economy. This Special Trust Account is open to any contribution from individuals as well as from legal, public, and private persons and entities. At the end of August 2020, this fund had reached more than US $3 billion, most of which was dedicated to support of economic activities (US $2.4 billion), while the rest went to the Ministry of Health for acquisition of medical equipment.

### Detection and Testing, Contact Tracing, and Isolation

In order to detect any cases from abroad at an early stage, a monitoring and surveillance system was set up at the start of the crisis in January 2020 at all entry points for international traffic. All passengers were systematically subjected to a temperature measurement by thermal camera and infrared thermometer in addition to a visual examination. Any traveler meeting the case definition had to be rushed to hospital for isolation and a sample for reverse transcription polymerase chain reaction (RT-PCR) examination. The case definition has been regularly updated to adapt to the evolving epidemiological situation. It takes into account symptoms for suspected cases and the real-time PCR test results for confirmed cases.

The capacity of PCR testing was initially limited to 3 laboratories and then has been gradually expanded to 38 laboratories. Two mobile laboratories under the INH were also mobilized, and 5 PCR laboratory platforms were installed in ships to provide tests for travelers between Morocco and European countries after reopening the borders to Moroccan citizens residing in foreign countries. A new circular from the Ministry of Health dated September 26, 2020 announced the availability of molecular screening tests by qRT-PCR for COVID-19 in all private laboratories in Morocco that meet the criteria in technical specifications.

As a result, the number of tests, which was very limited at the beginning, has gradually increased from around 100 per day to more than 160,000 tests per week.

Currently, home isolation is required for the majority of contacts especially for those without obvious symptoms. The duration of follow-up and isolation is set at 14 days from the last contact with a confirmed COVID-19 case [6].

### Organization of Case Management

The organization of the national response to COVID-19 has taken a series of rigorous measures concerning the management of cases affected by the disease. Among these measures is the management of all cases in a hospital environment. Thus, any case meeting the criteria of “possible case” or “confirmed case” was hospitalized in an isolation room. Severe cases were placed in an intensive care unit. Hospitalization capacity, which was very limited at the start, has been gradually increased through the establishment of field hospitals and capacity building of hospitals responsible for handling COVID cases.

Following the National Scientific, Technical and Advisory Committee’s recommendations, Morocco has decided to treat all patients with COVID-19 with hydroxychloroquine (HCQ) or chloroquine (CQ), combined with azithromycin (AZM) as first-line treatment and according to a standardized treatment regimen, in a systematic and structured manner. Thus, each confirmed case, even asymptomatic, received first-line treatment for 10 days [6]. The duration of first-line treatment can be extended by 5 days, before considering second-line treatment. Second-line treatment consisted of combination lopivinar/ritonavir for 10 days. Antibiotic therapy was prescribed only in case of a secondary bacterial infection. First-line treatment (HCQ or CQ + AZM) has been in effect in Morocco since the detection of the first cases, and it is still used now. These drugs are still available in pharmacies following the intervention of the Moroccan government with a subsidiary of the multinational producer.

Transfer to intensive care is done for severe cases according to pre-established criteria and after observation of the seriousness of the condition by the health care team.

At the beginning of September 2020, the Ministry of Health took new measures in the form of a memo [11] so that the treatment of potential cases can start as quickly as possible even before the release of PCR test results. Home care for asymptomatic or mild cases without risk factors has also been part of the treatment policy.

### Communication, Information, and Social Mobilization

Since the announcement of the epidemic in China, the Moroccan government has deployed an institutional and risk-based communication strategy. Different government officials, depending on their position and field of intervention, have followed one another to provide information on the epidemiological situation or measures taken. As soon as the first case was announced in Morocco, a daily press briefing on the situation linked to the epidemic was broadcasted live through national public television channels. With the increase in the number of positive cases later, the Ministry of Health reduced the frequency of the press briefing to 1 every 2 weeks.

Officials at the regional level as well as resource persons including scientists were also involved, in particular by appearing on official TV and radio channels during news bulletins and television or radio broadcasts.

Multiple awareness-raising spots on preventive measures have been produced and distributed continuously to raise awareness to avoid the risk of contamination. Leaflets have been prepared to educate travelers at points of entry.

### Lockdown and Lockdown Lifting

Given the exceptional nature of the situation related to COVID-19 and in accordance with the national constitution and regulations in force, Morocco declared a “state of health emergency” on March 19, 2020, allowing it to set up a series of preventive measures including lockdown with restriction of the movement of people and closure of national borders. In this context, the wearing of a mask was made mandatory. Reduction of the restrictive measures taken was later decided through a gradual lockdown lifting plan.

## SWOT Analysis

A SWOT analysis [12] was conducted to determine the strengths, weaknesses, opportunities, and threats related to the interventions carried out.

### Main Strengths

In this context, 9 major strengths deserve to be highlighted.

#### Preparation That Took Into Consideration the Lessons Learned From Other Public Health Emergencies of International Concern (PHEIC)

A pandemic preparedness and response plan was drawn up on the basis of the elements and orientation of the 2018-2022 National Health Security Plan that was implemented following the Joint External Assessment of the capacities required by the IHR (2005) and taking into account other response plans such as pandemic influenza, MERS-CoV, and Ebola disease.

The existence of know-how in the management of health crises and an awareness of the importance of developing the responsiveness of the health system in the face of a PHEIC was present, as recommended by the IHR (2005).

A risk assessment was established early by the Ministry of Health in the aftermath of the first signals of the COVID-19 epidemic that stressed that Morocco was at risk of being rapidly exposed to the disease. All interventions were carried out following precise knowledge of the level and origin of the risk.

Guidelines and procedures were gradually developed or revised, while adapting them to new scientific knowledge and the national epidemiological context and largely complying with WHO recommendations and guidelines.

#### Anticipated Reaction for Both Health and Financial Factors

A government action plan covering health, economic, and social aspects was implemented. A special fund was created at the initiative of the king of the country for the management of the pandemic. Programming and coordination of the actions of stakeholders were conducted to control the spread of the virus and its impact on economic and social life. All bodies of state and civil society were mobilized to ensure compliance with the measures recommended in the framework of the national response plan and the government action plan. Diplomatic missions were coordinated for exchange of expertise with the countries where the pandemic initially appeared. Financial aid measures were provided for vulnerable households and small businesses.

#### Proactive Epidemiological Surveillance and Notification of Cases Using an Electronic Platform

A fairly well-structured epidemiological surveillance system covering the entire national territory was present through structures dedicated to this function at national, regional, provincial, and prefectural levels and at border posts.

A pandemic surveillance system was established, which has benefited from the experience within the framework of the seasonal influenza surveillance system implemented gradually since 1996, which includes both clinical surveillance and virological surveillance of syndromes. An event-based surveillance system has been in place since 2018. A clear case definition has been constantly revised to adapt to the evolving epidemiological situation. Three telephone platforms were established for the management of alerts and referral of suspected cases. An interoperable and interconnected real-time electronic COVID case notification system was implemented, allowing data entry, analysis, and sharing at all levels.

#### Presence of Well-Trained Rapid Response Teams

Multidisciplinary and multisectoral teams were established for contact follow-up, coordinated by field epidemiologists or health professionals trained in epidemiology and rapid intervention. Contact tracing procedures were updated, with a view to their adaptation for the evolving epidemiological situation. The contact tracking system was reinforced with a mobile application called “Wiqaytna” based on Bluetooth technology, which allows notification of exposure to SARS-CoV2. Relentless contact tracing support has been provided by local and security authorities.

#### Increase in the Supply of Infrastructure, Equipment, and Health Products

Equipment was made available at all entry points, with modern temperature detection equipment (thermal cameras and remote thermometers). Hospital capacities were increased and reorganized, and patient reception conditions in the various COVID hospitals were improved. Military and civilian field hospitals were deployed to strengthen the health system in beds and equipment for intensive care and resuscitation. There was a significant increase in resuscitation beds and equipment. Production and industrial manufacture of masks, hydroalcoholic gel, and other disinfectant products were developed or reallocated, with price regulation. Capacity building of the laboratory system was conducted: Morocco had 4 laboratories at a biosafety level 3, which were used at the start of the epidemic and were subsequently reinforced until a good capacity was reached, including 30 laboratories with PCR platforms, 6 of which are mobile laboratories. Stocks of drugs, products, and personal protective equipment were constituted.

#### Patient Care in Accordance With Established Protocols

Management protocols were developed in collaboration with the Scientific, Technical and Advisory Committee of the Ministry of Health for the management of COVID-19 and were regularly updated based on new knowledge about the disease. Medicines and other pharmaceutical products were mobilized very early, and treatment services were integrated into all care structures and offered at home when the indication is justified. The organization of the care system and patient circuit were adapted in response to the new intervention logic. Free access to health care has been ensured for all suspected or confirmed cases. Psychologists were mobilized to provide psychological help to people weakened by illness and isolation. Several remote platforms were established to provide psychological support and counseling services to health professionals and citizens who develop certain disorders in the form of distress, depression, or acute panic disorders resulting from fear or confinement.

#### Solidarity Implication for Private Corporations

There has been exemplary compliance with barrier measures during the confinement period at the start of the crisis. Companies from the public and private sectors have supported the development of hospital services and consultation centers. There has been responsible involvement of certain private clinics in the management of COVID cases and in the management of other pathologies in the sense of relieving public hospitals and university hospitals. There have been massive amounts of participation by nongovernmental organizations (NGOs) and civil society organizations (CSOs) in various actions to fight COVID-19. Hotels and catering units have volunteered to offer reception rooms and catering services to convalescent patients or health personnel. University researchers were involved in the development of mathematical models to predict the spread of COVID-19 in Morocco. Manufacturers were involved in the production of masks and respirators. The ministry in charge of industry mobilized many companies within new business models that enable better production capacity.

#### Appropriate Governance and Coordination

Political commitment is present at the highest level of the state hierarchy (Royal commitment and of the whole government). There is a model of organization and coordination of the response that integrates all key sectors and takes into account all levels of intervention (central, regional, and local). There is a clear definition of the role and responsibilities of each ministerial department and other stakeholders including the business sector, the private sector, and civil society. The recently created Centre National des Opérations d’Urgence de Santé Publique (CNOUSP; National Public Health Emergency Operations Center) as part of the capacity building required by the IHR (2005) played a role as a focal points. Morocco already has a significant body of legislation and regulations to manage health crises in compliance with the law, which has been expanded during the COVID-19 pandemic. Ethical aspects were integrated in the policy and practices in terms of preparedness and response to the pandemic.

#### A Particular Interest of all Sectors in the Continuity of Essential Services

All sectors have an interest in maintaining vitally important activities during the confinement period based on all available staff resources and volunteers as well as regular monitoring of the supply or refueling of the markets by the availability of all necessities, food, hygiene products, or energy. Digitization of certain ministerial departments made it possible to guarantee the continuity of essential services by resorting to teleworking and by limiting the physical exchange of documents and administrative letters. Strengthening online banking services and the creation of a series of new digital services aimed to reduce the exchange of paper documents, thus limiting the risk of transmission of COVID-19. Practical manuals on teleworking in companies were published.

### Main Weaknesses

#### Governance and Leadership Were Sometimes Overtaken by Events 

Decision making was sometimes contested by the population and public opinion. Examples are decisions to confine certain towns in the following 6 hours, which precipitated part of the population towards an increased risk of accidents on overcrowded roads, or the decision to celebrate Eid El Adha (feast of sacrifice), which entailed a double risk of creating hotbeds of infection (contacts in uncontrollable cattle markets followed by extended family gatherings). There was a lack of collegial and socially appropriate decision making involving elected officials and the community. The multidisciplinary expertise that must characterize the composition and members of the scientific committee in a period of health crisis involving health, psychological, and social determinants was not considered with rigor. There was a lack of a clear strategy or procedures for involving NGOs. Directives and measures in the field of occupational health were implemented late and remained insufficient given the delay in strengthening this component.

#### Insufficient and Exhausted Human Resources

There was a lack of human resources even before the onset of COVID-19. It was difficult to fill the gaps in doctors and nurses, in particular for certain specialties and for resuscitators. It was also difficult to maintain and consolidate the commitment of health personnel due to the lack of clear motivation and a skills development program. The resources of the private sector, where nearly 50% of the physician workforce works, are underutilized to deal with the pandemic.

#### Delay in Communicating the Results of Diagnostic Tests

Despite the strengthening effort, the laboratory network was not large enough, and the results of biological tests were communicated with some delay. This had a negative impact on the surveillance process (test, trace, isolate) and precocity of treatment. Primary health care establishments (ESSP) were involved late in the management of COVID cases.

#### Management of Serious Cases Stifled by a Lack of a Sufficient and Quality Technical Platform

Cases admitted to intensive care units had high mortality. Conditions of stay in public hospitals were strongly criticized by patients. There were difficult working conditions in some hospitals.

#### Insufficient Communication to Increase the Confidence of the Population

Complex information management, given the impressive flow, was present, but there was also a considerable amount of fake news associated with the pandemic (very apparent infodemic). There was low perception of the seriousness of the epidemic by certain categories of the population. There has been a gradual decrease in compliance with the instructions transmitted relating to the application of barrier measures by a good segment of the population. Compliance with barrier measures has not been as strong as might have been hoped for given the quantity and intensity of preventive and incentive messages around COVID-19. Certain individuals wear unsuitable protective masks.

#### Difficulties in Managing the Business Continuity of Other Health Programs

There is a lack of a clear business continuity strategy for health programs in the context of the pandemic. Several basic health care structures have partially closed. There has been exclusive concentration of certain hospital services on COVID-19 as well as a significant reduction in health services and in the performance of other health programs.

## Opportunities

Morocco has all the assets to be able to take advantage of the current crisis linked to the COVID-19 pandemic by operating several levers at the same time while boosting public-private partnership and international cooperation with a view to reshaping the health system and ensure its resilience. Several opportunities are therefore offered and must be seized upon because of the lessons learned from the impact of the pandemic and the way it was managed.

Restructure the health system for strength and resilience as recommended in several initiatives and planning documents. Reconsider certain priorities of the health system and implement a new model of health development by giving more attention to the in-depth reform of the governance and functioning of the various health services.

Accelerate the implementation of the actions planned within the framework of the national health security plan including the establishment of a public health agency accompanied by a public health law as well as the development of a multirisk plan for management of all public health emergencies and humanitarian disasters. Take advantage of the reigning enthusiasm for effective strengthening of public-private partnerships. Seize the opportunities offered for the promotion of digital technology, teleworking, and telemedicine.

## Threats

The pandemic is much more than a health crisis: It is also an unprecedented socioeconomic crisis that has already had devastating social, economic, and political effects that will leave deep scars that will be slow to fade. Its threats to the health system and health security in general are numerous, 4 of which can have a lasting impact on the health system:

1. Risk of amplification of public health problems linked to other communicable diseases and noncommunicable diseases
2. Risk of a more acute installation of resistance to the directives and instructions of the authorities because of the “infodemic” that surrounds the pandemic via rumors and false information with no borders and is propagated at great speed by social media
3. Risk of loss of human resources due to contamination by the virus
4. Risk of a deep and uncontrollable saturation of case management structures

## Lessons Learned

During the first phases of the COVID-19 pandemic, Moroccans showed solemn commitment and collectively mobilized to face this PHEIC. The spirit that marked the whole society was animated by sincere patriotism, the spirit of sacrifice, as well as solidarity and loyalty. The response to the pandemic was distinguished by a strong political commitment and a mobilization of all segments of society: COVID-19 has revived a huge surge of solidarity.

The main lesson learned is a need to further strengthen national capacities to prepare for and respond to possible public health emergencies and to embark on a process overhaul of the health system, including research into innovative tools to ensure the continuity of the various disease prevention and control activities. In addition, the response to a health crisis is not the only responsibility of the health sector, and intersectoral collaboration is the guarantee of an optimal coordinated fight. Community-oriented approaches in public health have to be strengthened through more participation and involvement of NGOs and civil society in operational and strategic planning. Teleworking, telemedicine, and digitization emerged as one of the priority areas to be developed.

## Conclusion

Morocco is considered among the countries that got the virus under control early on, but when economic and social restrictions were eased, the number of cases increased considerably. The impact of the pandemic on the lives of citizens was obvious from all standpoints. One of the crucial lessons that can be learned is that the response to a health crisis not only is the responsibility of the health sector but also intersectoral collaboration is the guarantee of an optimal coordinated fight. Community-oriented approaches in public health have to be strengthened through more participation and involvement of NGOs and civil society in operational and strategic planning.

## Abbreviations

AZM: azithromycin
CNOUSP: Centre National des Opérations d’Urgence de Santé Publique
CQ: chloroquine
CSO: civil society organization
FETP: Field Epidemiology Training Program
HCQ: hydroxychloroquine
IHR: International Health Regulations
NGO: nongovernmental organization
PHEIC: Public Health Emergencies of International Concern
RT-PCR: reverse transcription polymerase chain reaction
WHO: World Health Organization
